# Is mammary not otherwise specified-type sarcoma with CD10 expression a distinct entity? A rare case report with immunohistochemical and ultrastructural study

**DOI:** 10.1186/1746-1596-8-14

**Published:** 2013-01-28

**Authors:** Guang-Zhi Yang, Jing Li, Hua Jin, Hua-Ye Ding

**Affiliations:** 1 Department of Pathology, The General Hospital of Beijing Military Command, Beijing, 100700, China; 2 Department of Pathology, The 263rd Hospital of PLA, Beijing, 101149, China

**Keywords:** Sarcoma, Breast, CD10, Undifferentiated sarcoma, Phyllodes tumour, Metaplastic carcinoma

## Abstract

**Abstract:**

Mammary sarcoma is extremely rare and the diagnosis is established only after metaplastic carcinomas and malignant phyllodes tumours are excluded. A rare case of not otherwise specified-type sarcoma with CD10 expression in the left breast in a 45-year-old female was presented. It was a high-grade tumour composed of spindle cells histologically. The immunohistochemical results showed that CD10, vimentin and EGFR were positive diffusely and SMA presented focally, whereas epithelial markers and other myoepithelial or myogenic markers were all negative. The electron microscope investigation demonstrated fibroblast-like features. The exact entity of the tumour remains to be studied because it resembles undifferentiated sarcoma or sarcomatoid metaplastic carcinoma to some degree, as well as high-grade malignant phyllodes tumour in particular.

**Virtual slides:**

The virtual slide(s) for this article can be found here: http://www.diagnosticpathology.diagnomx.eu/vs/9019879588725702

## Background

Mammary sarcomas are very rare and most display specified entities just like liposarcomas or angiosarcomas. There are still a few cases in the absence of specific differentiation, which were diagnosed of pleomorphic sarcoma or malignant fibrous histiocytoma and so on [1]. Leibl and Moinfar reported seven cases of not otherwise specified-type sarcoma with the most significant feature of consistent CD10 expression, which were designated as NOS-Type sarcoma with CD10 expression (NSCD10) and were considered as a variant of sarcomas with myoepithelial differentiation [2]. We also experienced one case of such sarcoma with diffuse and strong expression of CD10. Herein, we present the data of histology, immunophenotype and ultrastructure of the case and discuss in detail.

## Case presentation

A 45-year-old female presented with a giant mass in the left breast. The lump was first noticed by the patient herself six years ago. The tumour was only about 1cm in diameter at that time and grew slowly, thus no treatments were adopted. The tumour grew rapidly in the past half a year and its diameter reached 10 cm or so, therefore, the modified mastectomy and axillary lymphadenectomy were performed in our hospital.

**Figure 1:**
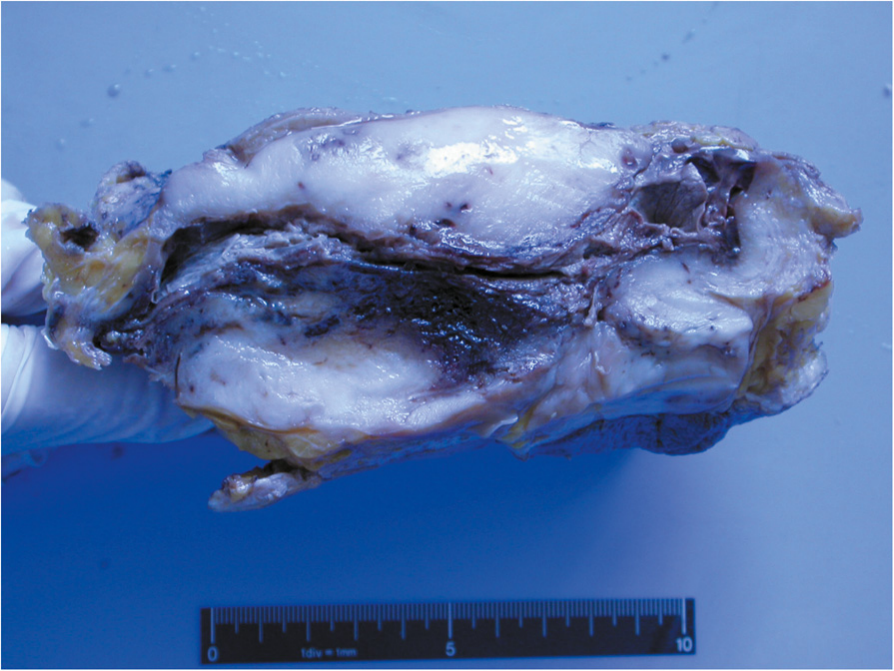
The cut surface was solid gray in fish appearance with haemorrhage and necrosis in the central area.

**Figure 2:**
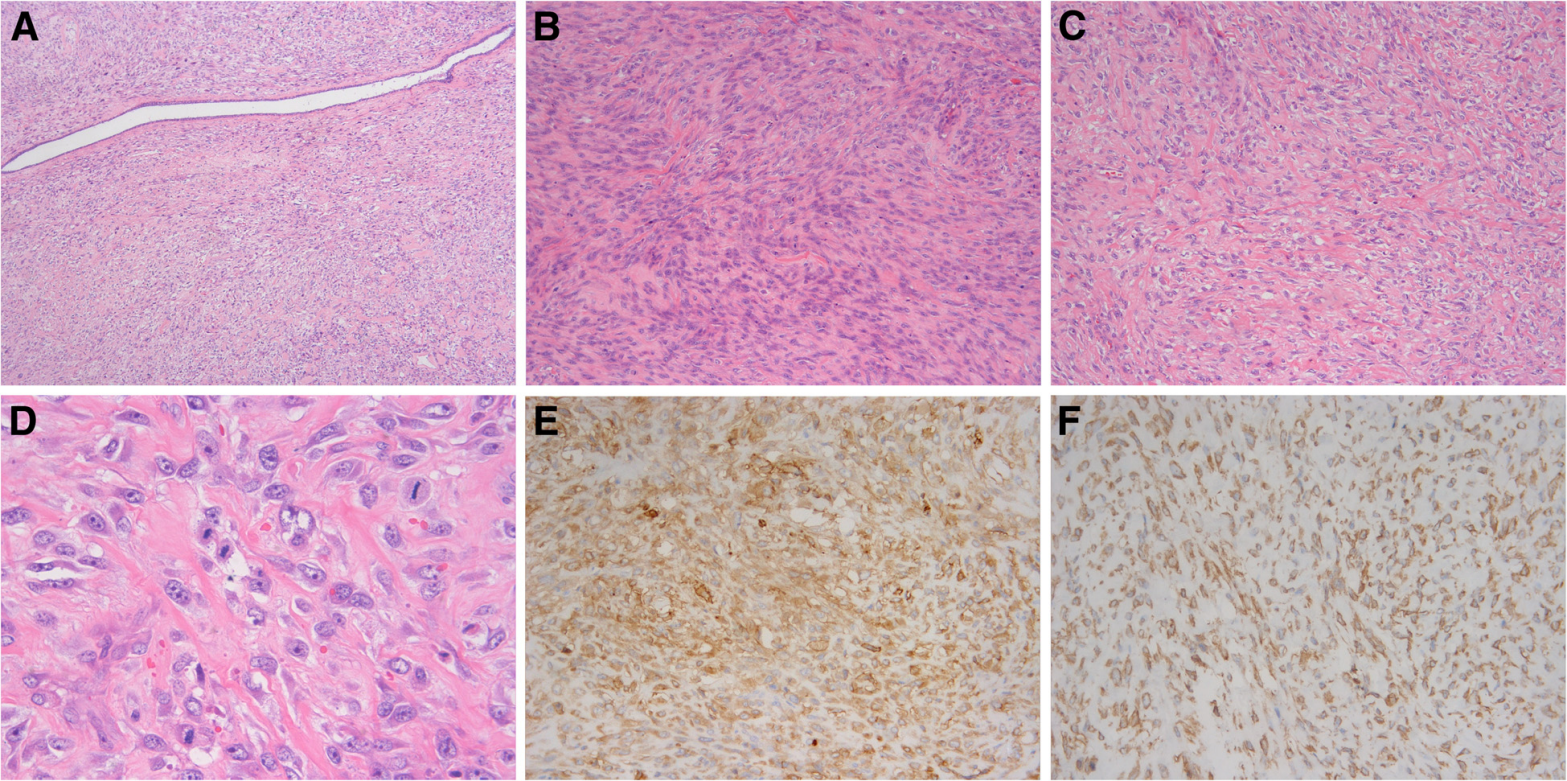
**A. The tumour was mainly composed of abundant spindle cells.** The tumour was absent of glands except that one elongated and narrow gland was displayed here after widely sectioned. (HE × 50) **B**. The tumour cells were gathered and abundant, whereas collagen was relatively rare. (HE × 100) **C**. Collagen bundles among the tumour cells were rather ample. (HE × 100) **D**. The tumour cells displayed inconspicuous eosinophilic or amphophilic cytoplasm, and striking and vesicular nuclei with coarse chromatin or obvious nucleoli. Mitoses were easily found. (HE × 400) **E**. CD10 was strongly and diffusely positive. (MaxVision method ×200) F. EGFR was strongly and diffusely positive. (MaxVision method × 200).

The tumour appeared a circumscribed neoplasm without envelopes, measuring 12 cm × 12 cm × 8 cm. The cut surface was solid gray in fish appearance with haemorrhage and necrosis in the central area (Figure 1). Under microscopic inspection, the tumour was found to be mainly composed of spindle cells interspersed with varying collagen bundles. The spindle cells were arranged in fascicles, occasionally in storiform pattern (Figure 2A). The collagen in most areas was scarce and inconspicuous (Figure 2B), whereas in some focal regions was rather ample (Figure 2C). Cytological observation showed that the cells displayed eosinophilic or amphophilic cytoplasm with ill-defined boundary. The nuclei were striking, polymorphic and vesicular with coarse chromatin or obvious nucleoli (Figure 2D). Mitoses were frequent and scored of 15–30 per 10 high power fields and atypical mitoses were easily found. The tumour invaded the around breast tissue. After sectioned widely, only one elongated and narrow gland was found (Figure 2A). None of the axillary lymph nodes was metastasized (0/18).

The immunostaining investigation showed that CD10 (56C6, Novacastra) and vimentin (V9, Invitrogen) were positive strongly and diffusely (more than 90% of tumour cells) (Figure 2E). Epithelial markers, such as panCK (AE1/AE3, Invitrogen), CK8/18 (NCL-5D3, Santa Cruz), CK7 (OV-TL 12/30, Dako), EMA (E29, Dako), and basal cell-type CKs including CK5/6 (D5/16B4, Invitrogen), CK14 (LL001, Santa Cruz), CK17 (E3, Dako) and high molecular weight CK (34βE12, Novacastra), were all negative. SMA (1A4, Santa Cruz) was focally positive (about 10%), and other myoepithelial or myogenic markers, including P63 (4A4, Santa Cruz), Calponin (CLAP, Novacastra), S-100 (S1-61, Santa Cruz), desmin (D33, Dako) and h-caldesmon (h-CALD, Santa Cruz), were negative. The tumour was also negative for CD34 (BI-3C5, Invitrogen), CD117 (1DC3, Invitrogen) and steroid receptors including ER (1D5, Invitrogen), PR (1A6, Invitrogen) and AR (H7507, Invitrogen). EGFR (H11, Dako) was membrane positive strongly and diffusely (more than 90%) (Figure 2F). Ki-67 (MIB-1, Dako) index was more than 70%.

**Figure 3:**
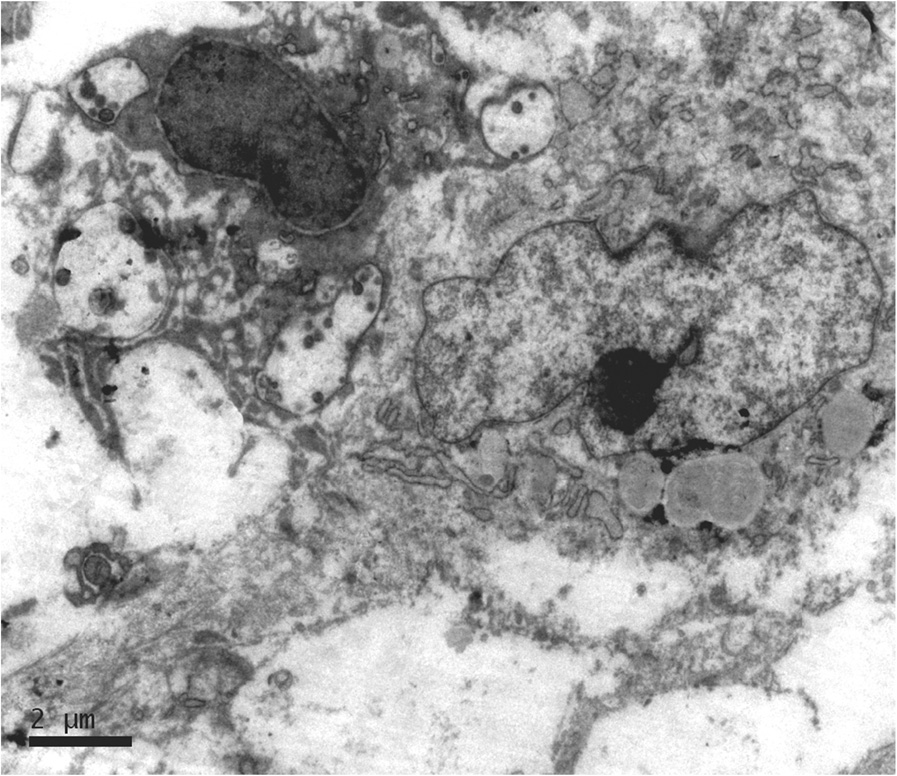
**The tumour cell was spindle and had the striking nucleus with irregular and distorted envelope and one significant nucleolus.** Apparatuses in cytoplasm were rare and lysosomes were relatively abundant.

Under perspective electron microscope observation, the tumour cell was spindle, arranged loosely, and no connection between cells was found. Apparatuses in cytoplasm were scarce and lysosomes were relatively abundant. The nucleus was striking with irregular and distorted envelope and one significant nucleolus (Figure 3). In short, the tumour cell exhibited fibroblast-like ultrastructure.

The patient was alive with follow-up for two years. Local recurrence was found seven months later and re-operation was performed.

## Discussion

In definition, mammary sarcomas are a heterogeneous group of malignant neoplasms that arise from the mammary stroma [1]. Excluded from this presentation are malignant lymphoid and hematopoietic tumours such as non-Hodgkin’s lymphomas, myelomas and granulocytic sarcomas, which have been rarely reported in the literature [3,4]. Sarcomas are extremely rare in the breast because most of previously diagnosed sarcomas, including some with obvious differentiation such as osteosarcoma or chondrosarcoma, were later proven to be sarcomatoid metaplastic carcinomas (MCs) due to modern immunohistochemical applications [1]. Therefore, the diagnosis of MCs must be excluded first by performing immunohistochemistry with adequate epithelial markers for suspected sarcomas in the breast pathology, as examined in our case with pan-CK, CK7, CK8/18 and basal-cell type CKs.

Among mammary sarcomas, a few do lack specific differentiation as reported by Leibl and Moinfar [2]. In histopathology, these tumours were composed of spindle cells with varying collagen fibrous matrix in most cases or myxoid matrix in a few. The spindle cells displayed highly polymorphic nuclei with numerous and frequent atypical mitoses, which confirmed the diagnosis of high-grade tumours. In immunohistochemistry, the tumours expressed CD10 consistently and therefore were denominated as NSCD10. NSCD10s were considered as a variant of sarcomas with myoepithelial features as indicated as expression of other myoepithelial markers including SMA, P63, CD29 and Calponin to a variable degree [2]. According to the criteria of histology and immunophenotype, our case was also supposed to be diagnosed of NSCD10.

The perspective electron microscope observation was also performed in our case. In agreement with the negativity of all epithelial markers, no evidence of epithelial differentiation was found. However, though CD10 was strongly and extensively positive, no evidence of myogenic differentiation was found either. These results also support the opinion that none of available so-called myoepithelial markers is specific for myoepithelial cells until now. It is a pity that electron microscope investigations had not been included in the report of Leibl and Moinfar [2]. Since myofibroblastic features are generally more easily documented by electron microscope rather than immunohistochemistry in poorly differentiated sarcomas, our case seemed more to fit into an undifferentiated sarcoma. Thus, it put forward a question whether NSCD10s were actually one type of undifferentiated sarcomas.

In the process of diagnosis, malignant phyllodes tumour (PT) had ever been taken into account since the tumour demonstrated atypical leaf-like feature locally. While PTs usually display leaf-like biphasic structure, some cases with obvious stromal overgrowth may lack it, especially high-grade or recurrent PTs. In addition, the feature of the patient’s history, which suggested a probably benign or borderline lesion at first and then sarcomatous overgrowth, is one of common phenomena in malignant PTs. In this situation CD34 is a valuable marker as PTs are frequently positive for CD34 [5,6]. The negativity of CD34 in this case was one of the strong evidences against diagnosis of malignant PTs.

Stable expression of CD10 is one of the most striking features of NSCD10s. CD10, also called common acute lymphoblastic leukemia antigen (CALLA), initially recognised to be expressed by lymphoid precursor cells and some B cells, and mainly applied in diagnosis of haematological malignancies. CD10 is also now used for diagnosis of various non-haematological neoplasms, such as endometrial stromal tumours, renal cell carcinomas. In breast, CD10 is often adopted as a marker for myoepithelial cells in differential diagnosis [7]. Recently, the role of CD10 has been noticed in diagnosis and prognosis of PTs. A progressive and significant increase of stromal cell CD10 expression in mammary fibroepithelial lesions with the ability to metastasis had been reported by two groups respectively [8,9]. Among all types of fibroepithelial lesions, expression of CD10 was the greatest in malignant PTs, in which the positive cells ratios were about 20% and staining intensity was moderate to strong. The expression of CD10 in our case was much more extensive and intensive than those of malignant PTs, which was another proof against diagnosing PTs. Given that CD10 is a cell surface neutral endopeptidase, one member of metalloprotease family, its role of facilitating malignant potential would be explained [8], which was also supported in the breast and colorectal carcinoma [10,11]. It is reasonably inferred that NSCD10s may rank the top of PTs if they were seen as a continuous spectrum since an increasing trend in CD10 expression with malignancy grade in fibroepithelial neoplasms.

The further differential diagnosis comprised of leiomyosarcoma and myoepithelial carcinoma (MEC) because of similarity in morphology or immunophenotype. Leiomyosarcomas may exhibit some morphological features associated with NSCD10s, for example, spindle cells with elongated nuclei with blunt ends. However, leiomyosarcomas usually do not, or only focally express CD10. And desmin or h-caldesmon are very useful markers for differential diagnosis, which are positive in leiomyosarcomas and negative in NSCD10s. MECs entirely composed of spindle cells have been reported, which would cause differential diagnostic problems because CD10 is diffusely positive too [12]. But MECs also express other myoepithelial markers extensively, such as p63, Calponin and SMA and should express CK5/6 or CK5 which are not expressed by NSCD10s.

Frequent expression of EGFR (epidermal growth factor receptor, also named as Her-1) is another feature of NSCD10s. EGFR is a valuable marker in combination with ER, PR, Her-2 and CK5/6 to identify basal-like breast carcinomas in genetic and molecular classification, which most MCs belong to [13-15]. The expressions of EGFR and myoepithelial markers in NSCD10s are quite similar to those in sarcomatoid MCs, suggesting an intrinsic link between them [16]. With the immunophenotype from carcinomatous type to sarcomatous type according to decrease of CKs expression, the biological behaviour of MCs has a tendency from carcinoma to sarcoma too. It is assumed that NSCD10s represent the extreme sarcomatous end of MCs with myoepithelial differentiation scale. Recent studies also proposed that CD10 was one of useful markers to track stem cells in the breast carcinoma, especially precursors to MCs [17,18]. In the opinion that the tumour arises from stem cell, MCs are supposed of bi-directional differentiation to epithelium and myoepithelium, and NSCD10s are rather bi-directional differentiation to mesenchyma and myoepithelium.

High EGFR expression in NSCD10s also meets a potential for molecular target therapy, which has been verified in some solid tumours, such as colorectal cancer, non-small-cell lung cancer and squamous cell carcinoma of the head and neck [19-21]. However, maybe just as in breast cancer, EGFR mutation or amplification were rare events and membranous staining pattern of EGFR might be the best way to decide eligibility for anti-EGFR therapy, target therapy was valuable in NSCD10s [22]. It is interesting to know if gene mutation or amplification of EGFR would be detected in this case.

The origin of NSCD10s still remains to be known. Mammary sarcomas are traditionally thought to arise from interlobular mesenchymal elements, which comprise the supporting mammary stroma. For example, angiosarcoma, one of the most common forms of mammary sarcoma, is thought of endovascular origin [23,24]. However, as far as mammary sarcoma with bone and cartilage is concerned, it is difficult to explain because of absence of the counterpart. It is even a big challenge to infer the origin of sarcomas comprising two or more unrelated differentiated tissue elements, such as malignant mesenchymal tumour reported in the bladder [25]. Now it is believed that sarcoma originates from primitive cell with the capacity for totipotent differentiation. So it is proposed that NSCD10 arises from the stem cell and is differentiated to myoepithelial cell to some degree, but the histogenesis remains uncertain. Further studies dealing with molecular features of NSCD10 are valuable to help elucidate the origin of NSCD10 together with MC and PT because of their close relations.

## Conclusion

In summary, we reported a rare case of NSCD10 that is characterized of extensive and intensive expression of CD10 in the breast. In our opinion, whether the entity of the high-grade tumour is distinct remains unclear because of resemblance to undifferentiated sarcoma or sarcomatoid MC to some degree, as well as top-grade malignant PTs in particular. Further studies should be performed to elucidate the exact entity of NSCD10, especially dealing with the molecular properties, which will also deepen knowledge of the relevant tumours.

### Consent

Written informed consent was obtained from the patient for publication of this case report and any accompanying images. A copy of the written consent is available for review by the Editor-in-Chief of this journal.

## Abbreviations

NSCD10: Not otherwise specified-type sarcoma with CD10 expression; MC: Metaplastic carcinomas; PT: Phyllodes tumour; CK: Cytokeratin; EMA: Epithelial membrane antigen; SMA: Smooth muscle actin; EGFR: Epidermal growth factor receptor; ER: Estrogen receptor; PR: Progesterone receptor; AR: Androgen receptor; MEC: Myoepithelial carcinoma.

## Competing interests

The authors declare that they have no competing interests.

## Authors’ contributions

GY collected information and wrote the manuscript. JL performed histological and immunohistochemical investigation. HJ performed electron microscope investigation. HD made the pathological diagnosis. All authors read and approved the final manuscript.
